# Physical and clinical results of a radiation bra in patients treated with total skin electron beam therapy

**DOI:** 10.1016/j.phro.2024.100628

**Published:** 2024-08-16

**Authors:** Isabel Falke, Khaled Elsayad, Mohammed Channaoui, Christian Kandler, Christos Moustakis, Hans Theodor Eich

**Affiliations:** a Department of Radiation Oncology, University Hospital Munster, Munster, Germany; b Department of Radiation Oncology, University Hospital Basel, Switzerland; c Department of Radiation Oncology, University Medical Center Leipzig, Leipzig, Germany

**Keywords:** Mycosis fungoides, Breast, Boost, TSEBT, Upright radiotherapy

## Abstract

Total skin electron beam therapy (TSEBT) in female patients with large or pendulous breasts is usually associated with shaded inframammary folds. In this analysis, 18 patients with cutaneous malignancy and pendulous breasts were irradiated with a radiation bra and five patients received TSEBT without bra. All patients had moderate or severe sagging of the breasts. The median inframammary dose in the radiation bra group was 89% of the prescription dose versus 4% in the group without bra. The usage of the radiation bra enables an adequate radiation dose for the inframammary folds during TSEBT with no additional local irradiation.

## 1 Introduction

Total-skin electron beam therapy (TSEBT) is an effective treatment approach for various skin malignancies [1], [2]. During TSEBT, patients are treated upright to receive more homogenous doses to the skin surface [3], [4]. Local supplementary radiotherapy (RT) is often applied to underdosed anatomic sites such as inframammary folds in patients with pendulous or large breasts [4]. Supplemental radiotherapy to inframammary folds may be associated with under- or overdosed radiation field junctions. To dispense supplemental local RT to inframammary folds, radiation oncologists try to expose these shaded regions to radiation during TSEBT to reduce cancer patient's visits to hospitals for additional inframammary radiotherapy [5]. Radiotherapy was successfully delivered without attenuation of treatment beams using a radiation bra [6].

The objective of this study was to examine the potential dosimetric and clinical advantages of a new radiation bra. Additionally, we compared the inframammary radiation therapy dose distributions to validate the assessment for patients undergoing TSEBT with a control group treated without the bra.

## 2 Materials and methods

**Fig. 1:**
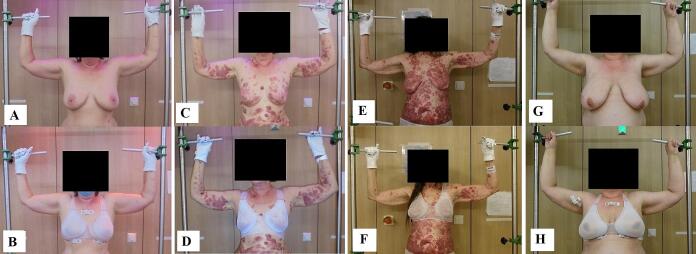
Four patients with mycosis fungoides, depicted with (lower images) and without (upper images) a radiation bra, show moderate sagging of the breasts [patients 1 (A-B), 5 (C-D), and 6 (E-F)] and severe sagging of the breasts [Patient 15 (G-H)].

Twenty-three females with advanced-stage cutaneous lymphoma were treated at our department between September 2019 and January 2024 (as part of the prospective observational study S-MISR; German Clinical Trials Register number: DRKS00030375). Eighteen patients received TSEBT treatment using the six-dual-field technique [3], [4] with the Chabner XRT® bra and five patients without. Nineteen patients had histologically confirmed mycosis fungoides (MF) stage IB to IV with inframammary lesions. Three patients had Sézary syndrome (SS) and one patient had diffuse marginal-zone lymphoma. According to Regnault breast ptosis classification [7], patients had moderate (N = 10) or severe (N = 13) sagging of the breasts (Fig. 1). Nine patients had inframammary patches/plaques, nine patients had tumors, and five patients had erythroderma. Thirteen patients were treated with ultra-hypofractionated TSEBT regimen (2 × 4 Gy), eight patients received 8 × 1.5 Gy to a total dose of 12 Gy, and two patients received higher radiation doses (24 and 30 Gy, respectively). All subjects were treated in a standing position for delivering TSEBT [3]. In the group without bra, patients received supplemental radiotherapy to the inframammary folds with 8–12 Gy in a fraction dose of 2 Gy after TSEBT.

The radiation bra used was composed of 92 % nylon and 8 % spandex, with a thickness is 1.1 mm. The material does not significantly alter the radiation beam properties of 6 MeV electrons used in TSEBT [8].

As a quality assurance procedure, we performed transmission and attenuation measurements on the Alderson phantom. The dosimetric verification (transmission, attenuation) measurements of the material (Chabner XRT® garment) has been reported. The bra offers proper reproducibility with a mean displacement of approximately 1 mm and 1° for all directions [9]. TLD measures were acquired for 10 patients utilizing TLD-100 rods. The readout was done using a Harshaw 5500 computerized TLD-Reader. All TLDs were prepared for measurement by placing them in a TLD PTW annealing oven heats at 400 °C for ten minutes and then left to cool to room temperature. During the first radiation fraction, dosimeters were taped to the patient's skin surface at the inframammary folds. As a reference, additional measurements on the sternum and thoracic spine were performed. A hot nitrogen gas stream warmed each dosimeter to 135 °C for 15 s to reduce the fading effect. After the temperature was raised to 240 °C, the light emission related to the dosimeter's absorbed dose was calculated and documented as a percentage of the prescribed radiation dose. Fifteen TLD measurements were performed on 10 patients' skin surfaces (five in the radiation bra group and five patients without bra). As a reference of the delivered radiation dose, we measured the radiation doses on sternal surface and vertebral 6th thoracic spine.

## 3 Results

**Fig. 2:**
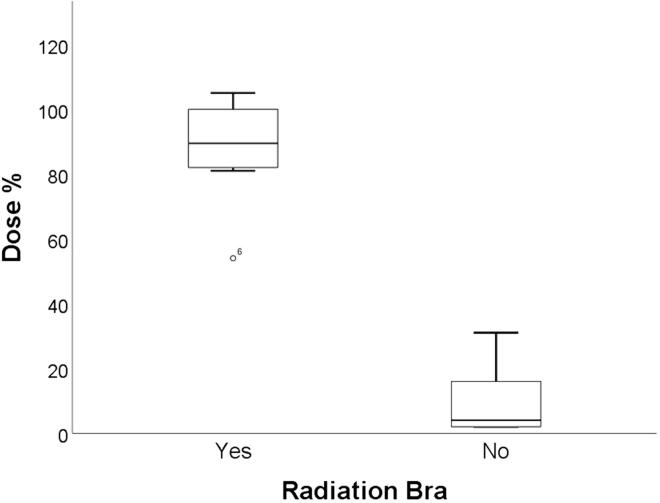
Boxplot shows the percentage of inframammary radiation dose (of the prescription dose) in the radiation bra group (N = 10) compared to the group without a bra (N = 5).

The dosimetric results were available for five patients in the group wearing bra (N = 10) and five in the group not wearing bra (N = 5). In the whole cohort, the median sternal dose was 92 % (range: 71–112 %), and the median back dose (at the level of the fourth thoracic vertebra) was 104 % (range: 70–108 %). In the radiation bra group, the median inframammary dose was 89 % (range: 54–105 %) compared to 4 % without wearing a bra (range: 2–31 %). For the control group, patients with moderate breast ptosis (N = 2) had median inframammary doses of 16 % and 31 % of the prescribed dose. Patients with severe ptosis (N = 3) had median inframammary doses of 2 % (range: 2–4 %) of the prescribed dose (Fig. 1 **and** Fig. 2).

All patients in the radiation bra group experienced a clinical response of the inframammary cutaneous lesions without local radiotherapy (Supplementary Table S1 and Figure S1). At a median follow-up period of eight months (range: 3–45), two patients (11 %) in the radiation bra group experienced local inframammary relapses and received salvage local radiation. We could not detect substance dependency with the radiation bra or any effect on the radiation dose distribution (Supplementary Table S2).

## 4 Discussion

This technical note presents the initial assessment of a radiation bra in TSEBT. The study revealed the following key findings: Firstly, utilizing a breast bra ensures an adequate distribution of dose to the inframammary area, thereby helping patients with pendulous breasts avoid under-treatment of the inframammary fold. Secondly, it may be possible to omit supplemental local radiotherapy in this area to reduce the number of radiation sessions and associated time toxicity. TSEBT in the upright position is a practical treatment option for patients with widespread skin manifestations. However, the dose distribution in the inframammary area is often insufficient for patients with pendulous breasts, leading to potential underdosing [4]. Furthermore, previous investigations indicated a relationship between dosimetric accuracy and patient weight, as well as dosimetric accuracy and gender [8], [10], [11], [12].

Based on our results, we further recommend the usage of a radiation bra in female patients receiving TSEBT. These findings from a feasibility study will help in planning a more thorough investigation into the potential correlation between bra size and dose measurement results. Nevertheless, the information presented offers essential data for optimizing dose allocation during TSEBT. There's a need for international guidelines to standardize TSEBT techniques and quality assurance procedures across different treatment centers.

The limitations of the current analysis include the small sample size, the limited number of measurements conducted for each patient, the retrospective nature of this analysis, and uncertainty in the placement of the TLDs. TLD measurements are essential for quality assurance in female patients undergoing TSEBT. Caution should be exercised when interpreting our findings as the patients we examined had very large breasts or were obese. In conclusion, the use of radiation bras enables an adequate radiation dose for the inframammary folds and a low rate of inframammary recurrence during TSEBT. Therefore, supplementary local radiotherapy in this area may be omitted to minimize the number of radiation sessions and the associated time toxicity.

## Funding information

This research received no funding.

## CRediT authorship contribution statement

**Isabel Falke:** . **Khaled Elsayad:** Conceptualization, Methodology, Software, Data curation. **Mohammed Channaoui:** Conceptualization, Methodology, Software. **Christian Kandler:** Data curation. **Christos Moustakis:** Data curation. **Hans Theodor Eich:** Conceptualization, Methodology, Software.

## Declaration of competing interest

The authors declare that they have no known competing financial interests or personal relationships that could have appeared to influence the work reported in this paper.
