# Supplementary material for: Physical and clinical results of a radiation bra in patients treated with total skin electron beam therapy
Source: Phys Imaging Radiat Oncol. 2024 Aug 16;31:100628. doi: 10.1016/j.phro.2024.100628 (PMC11381991; doi:10.1016/j.phro.2024.100628)

Supplementary Table S1: Patients characteristics

Patient	Diagnosis/ Clinical stage	Type of submammary lesion	Breast ptosis classification	Total TSEBT dose/fraction dose (Gy)	Overall skin response	Inframammary skin response	Inframammary local control (months)
**Patients treated with radiation bra**							
**1**	MF/IIB	Plaque and tumors	Moderate	8/4	CR	CR	45
**2**	MF/IIB	Plaque and tumors	Severe	12/1.5	PR	CR	2
**3**	SS	Patch	Moderate	12/1.5	PR	CR	26
**4**	MF/IB	Patch	Severe	12/1.5	PR	CR	35
**5**	MF/IIB	Plaque and tumors	Moderate	12/1.5	CR	CR	39
**6**	MF/IIB	Plaque and tumors	Moderate	8/4	PR	PR	3
**7**	MF/IIB	Plaque and tumors	Severe	12/2	PR	CR	23
**8**	MF/IIB	Plaque and tumors	Severe	8/4	CR	CR	4
**9**	SS	Erythrodermia	Moderate	12/1.5	CR	CR	8
**10**	MF/IIB	Plaque and tumors	Moderate	8/4	PR	PR	8
**11**	MF/IIB	Plaque and tumors	Moderate	8/4	PR	CR	7
**12**	MZL	Plaque	Severe	8/4	CR	CR	7
**13**	MF/IB	Patches and plaque	Severe	8/4	SD	SD	6
**14**	MF/IV	Erythrodermia	Severe	8/4	PR	CR	24
**15**	MF/IB	Plaque	Severe	8/4	CR	CR	30
**16**	MF/IIB	Plaque and tumors	Moderate	8/4	CR	CR	4
**17**	MF/IB	Patches and plaque	Moderate	8/4	PR	CR	4
**18**	MF/IIB	Plaque and tumors	Severe	8/4	PR	CR	4
**Patients treated without radiation bra**							
**19**	SS	Erythrodermia	Severe	30/2	SD	SD	4
**20**	MF/IIB	Plaque and tumors	Moderate	12/1.5	SD	SD	3
**21**	MF/IB	Plaque	Severe	12/1.5	PR	PR	24
**22**	MF/IIB	Plaque and tumors	Severe	12/1.5	PR	CR	24
**23**	MF/IV	Erythrodermia	Severe	24/1	PR	CR	6

MF: mycosis fungoides, SS: Sezary syndrome, CR: complete remission, PR: partial remission, SD: stable disease

Supplementary Table S2: The TLD measurements on the Alderson Phantom were compared with and without the radiation bra

Dosimeter	Site of dosimeter	Dose without Bra (%)	Dose with Bra (%)
First layer			
1	Right	99%	106%
2		103%	104%
3	midst	70%	79%
4		102%	99%
5	Left	114%	113%
Second layer			
6	Right	123%	121%
7		120%	117%
8		118%	114%
9	midst	113%	110%
10		116%	115%
11		121%	118%
12	Left	125%	122%
Right breast			
13	cranial	8%	5%
14	cranial medial	2%	2%
15	caudal medial	6%	5%
16	caudal	14%	13%
17	caudal lateral	12%	13%

Supplementary Figure S1: Postradiation images show complete remission of skin manifestations 8 months after total skin electron beam therapy (TSEBT) in patient 5 (A) and partial remission of skin lesions 4 weeks after TSEBT in patient 6 (B).

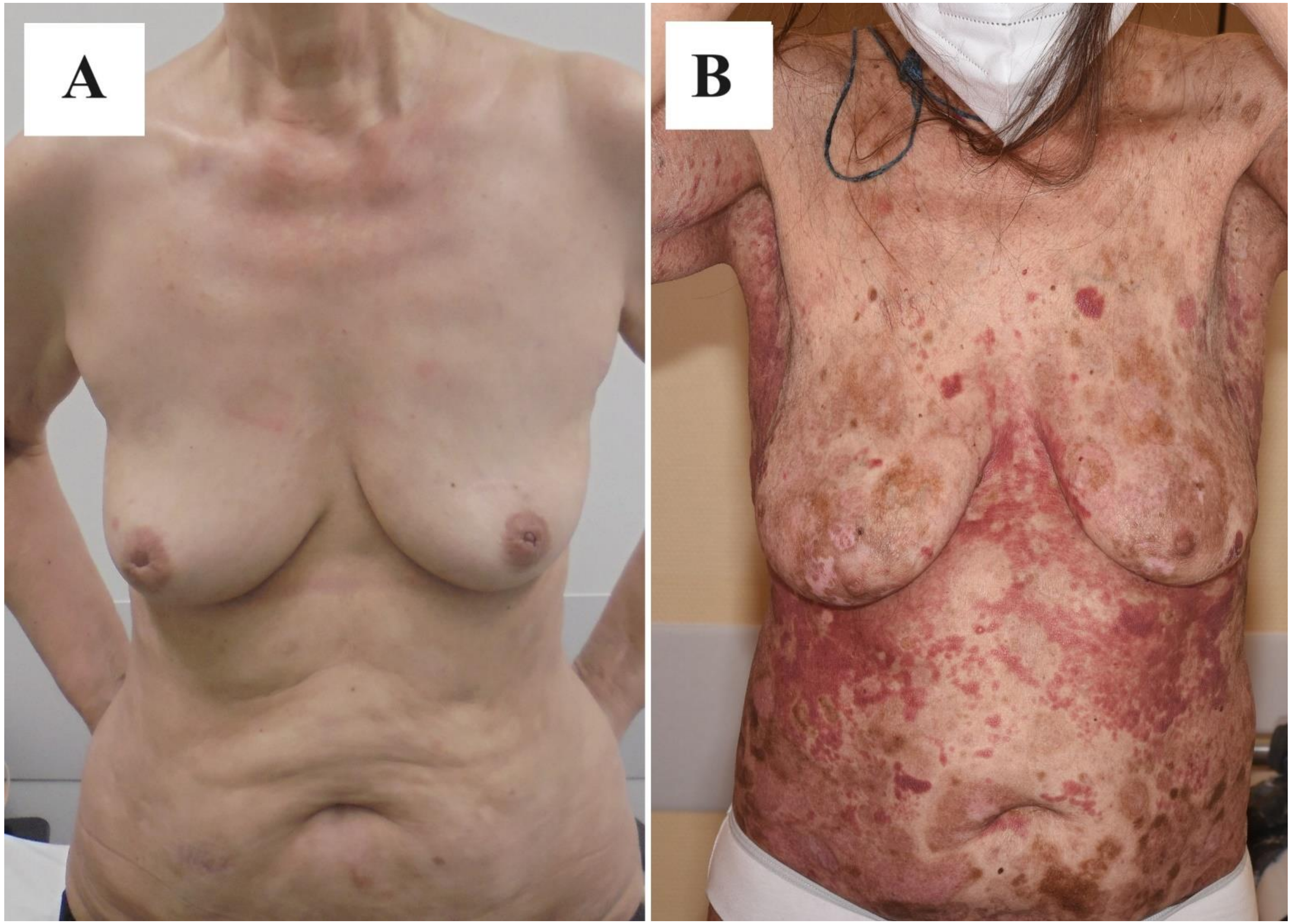

Supplement: Supplementary Data 1 [file mmc1.pdf]
